# MINDING THEIR OWN BUSINESS: MARRIED WOMEN AND CREDIT IN EARLY EIGHTEENTH-CENTURY LONDON*

**DOI:** 10.1017/S008044011500002X

**Published:** 2015-12

**Authors:** Alexandra Shepard

## Abstract

Taking a micro-historical approach, this paper explores the business activities of Elizabeth Carter and Elizabeth Hatchett, two married women who operated together as pawnbrokers in London in the early decades of the eighteenth century. Based on a protracted inheritance dispute through which their extensive dealings come to light, the discussion assesses married women's lending and investment strategies in a burgeoning metropolitan economy; the networks through which women lenders operated; and the extent to which wives could sidestep the legal conventions of ‘coverture’ which restricted their ownership of moveable property. It is argued that the moneylending and asset management activities of women like Carter and Hatchett were an important part of married women's work that did not simply consolidate neighbourhood ties but that placed them at the heart of the early modern economy.

This paper takes a micro-historical approach to reflect on processes with macro-historical consequences, focusing on a case-study of women's lending activities during a period of rapid commercial development. The discussion centres on the enterprise of two married women – Elizabeth Carter and Elizabeth Hatchett – who operated together as pawnbrokers in London in the early decades of the eighteenth century. A protracted inheritance dispute over Carter and Hatchett's property, during which their extensive dealings were detailed by many witnesses, allows an unusual glimpse, first, of the networks through which female lenders operated, and, secondly, of the ways they sidestepped the legal conventions of coverture which (in theory) restricted married women's ownership of and contractual rights to moveable property. The paper concludes with some tentative suggestions of how we might, on the basis of this case, rethink married women's position in the early eighteenth-century economy.

In recent years there has been growing recognition among historians of the roles played by women's business activities in the expanding urban economies of eighteenth-century Britain, just as women's labour force participation has been identified by Maxine Berg as critical to the pace and character of industrialisation.^1^ Jan de Vries has similarly cited women's ‘industriousness’ as a determinant of the growing demand that provided the stimulus for industrialisation, which, he claims, was linked to their aspirations for an expanding range and volume of consumer goods.^2^ In this latter case, women's roles as consumers are arguably privileged above their contributions as producers. In addition, while social emulation has been effectively rejected as the driving force shaping women's consumption habits, there remains a historiographical tendency to construct women's consumption patterns in terms of taste, identity and desire, rather than as a form of economically driven investment.^3^ My discussion here draws out the links between credit, consumption and investment, recasting consumption as a form of asset management in order to identify a further dimension of married women's work that formed a critical component of the eighteenth-century economy.

Not least because of the legal restrictions curtailing married women's property rights, wives’ lending activities are extremely difficult to discern since they rarely generated any formal legal record.^4^ By contrast, the credit extended by single and widowed women is becoming increasingly recognised by historians as a significant feature of the early modern English economy.^5^ Amy Erickson has even argued that the cash supplied by single women provided the necessary capital investment underpinning England's distinctive trajectory of economic development, thereby attaching causal significance to single women's lending as a driver of change.^6^ Married women's involvement in brokering credit, by contrast, is often represented not only as hedged by legal constraints but also as ‘informal’, and principally associated with networks of female solidarity. Small scale and short term, the lending and borrowing undertaken by married women might readily be cast as part of the support networks that enabled households to get by in the face of haphazard income streams and a limited cash flow.^7^ Married women's credit relations therefore tend to be approached by historians (if acknowledged at all) as necessitated by a relatively primitive economy rather than possessing any other discernible rationale, suggesting a substantial disparity in women's economic agency on the basis of their marital status that was, moreover, in contradiction of the *social* authority ascribed to married women in relation to their single counterparts.^8^

This paper rejects any straightforward characterisation of married women's credit broking as relatively marginal or solely inspired by mutual reciprocity. Instead, it is argued here that married women's borrowing and lending activities stemmed from their responsibilities for asset management within their households, which were understood as a form of enterprise. In the early modern period, a good deal of most people's wealth was stored in household goods and literally vested in clothes – those items over which women, and especially married women, tended to exercise responsibility. Anachronistic concepts of wealth, which prioritise money above other forms of moveable wealth, risk producing too narrow an approach to early modern consumption patterns. The consumption of goods represented investment strategies rather than simply ‘spending’, enmeshed within and enabling complex credit relations in which married women were crucial brokers. Just as historians have questioned expectations that rituals of childbirth in the early modern past were inspired by sisterhood, so we might also problematise assumptions that women lenders were more likely to privilege solidarity over self-interest.^9^ Married women's responsibilities for saving and accounting for household goods placed them at the heart of the early modern economy – in terms of the daily workings of exchange – rather than at its margins. The case of Elizabeth Carter and Elizabeth Hatchett suggests that credit broking could establish some married women as important patrons within their neighbourhoods and beyond, and could also present them with significant business opportunities.

We might interpret the agency of women like Elizabeth Carter and Elizabeth Hatchett in terms of ‘resistance’ to patriarchal norms, not least since their business dealings involved adept negotiation of the legal constraints associated with coverture – that is, the common law expectation that assigned ownership of a woman's moveable property to her husband and that denied married women the right to enter in to contractual relations of debt and credit.^10^ However, rather than exclusively constructing female economic agency in terms of resistance, there is also a case for questioning the extent to which such norms pervaded everyday life.^11^ What we may, in fact, be glimpsing in this case is a set of routine expectations that married women held a major and active stake in household enterprise, to which they might contribute independently as well as in partnership with a spouse.^12^ The attraction of such an interpretation lies not least in the prospect it affords of an expanded approach to women's economic agency as *productive* of the economic landscape and not simply as *responsive* to its constraints.

Elizabeth Carter and Elizabeth Hatchett's activities were at the centre of a testamentary dispute heard in the bishop of London's Commissary Court between October 1723 and January 1726. The case concerned the estate of Elizabeth Carter, of St Stephen Coleman Street, who had died of a fever in 1722.^13^ Described as a midwife, Carter had also been involved in moneylending and pawnbroking, letting rooms, leasing houses and selling tobacco. Carter was a widow when she died, having been predeceased about two years previously by her husband, Humphrey, who had been a baker. The litigation concerned Hatchett's claims to Carter's goods, which were in dispute following Hatchett's own death ‘of Jaundice’ in June 1723, a mere nine months after Carter's death.^14^ The parties in dispute were a young singlewoman, Eleanor Jennings, who at the age of twenty-three was executrix and primary beneficiary of Hatchett's will, and Mary Lucas (wife of Samuel Lucas), who was Elizabeth Carter's sister. Eleanor Jennings was the eldest of three daughters belonging to a neighbouring couple of Elizabeth Carter and Elizabeth Hatchett. Mary Lucas sued Eleanor Jennings on the grounds that Elizabeth Hatchett had wrongfully bequeathed goods to Jennings that had belonged to Elizabeth Carter and to which Hatchett had no claim, and which therefore rightfully belonged to Lucas.^15^

The case revolved around the working relationship between Elizabeth Carter and Elizabeth Hatchett who had clearly had a long association for up to two decades before Carter's death. In dispute was whether they had been business partners, equally bound to each other by intimacy and friendship (on the one hand), or (on the other hand) whether they had been in each other's debt. Thirty-nine witnesses were produced to give evidence (twenty-three women and sixteen men), some of whom were called on more than once, in a case which lasted for more than two years. The case was unusually drawn out, and the number of witnesses well exceeded the average (which was between six and seven) established from a sample of testamentary disputes heard by the same court between 1700 and 1728.^16^ Since both Carter and Hatchett had died without any direct heirs (neither had any surviving children), at stake were the relative claims of Carter's sister and Hatchett's legatees to an indeterminate quantity of moveable property.^17^ Items mentioned frequently in the witness testimony were a gold striking watch, a gold chain, a diamond brooch, leases to tenements, lottery tickets and varying amounts of cash and goods.

In support of Mary Lucas's case against Eleanor Jennings, Carter was represented by the majority of witnesses as an extremely wealthy and successful woman (both while a wife and as a widow), on whom Hatchett had depended as a servant, and whose extensive goods Hatchett had misappropriated during Carter's final sickness and after her death. The evidence in support of Eleanor Jennings's claims instead focused on the extent to which the women had worked in *partnership* with each other – with Hatchett as the senior partner – and stressed Carter's longer-term dependence on Hatchett for her basic maintenance and for her care in her final sickness. The dispute therefore produced divergent accounts of the women's relationship and the women's fortunes, which serve as a reminder that witness testimony was heavily shaped by the competing claims that on the one hand undoubtedly exaggerated Carter's wealth and success and that on the other hand stressed Hatchett's self-sufficiency and Carter's obligation to her. The details provided by witnesses in the case nonetheless testify to what was *imaginable* concerning these two women's dealings, even if we will never know which aspects of their relations were ‘true’.^18^

The volume of testimony in this case (amounting to more than 100 folios of depositions alone) provides extraordinary insight into forms of work that married women routinely undertook, which involved asset management and credit broking.^19^ This is evident not only from the details of Hatchett and Carter's dealings but also from the varied use of their services by a number of married women. The case also illustrates the extent to which married women were able to circumvent the legal constraints of coverture. The extensive, and often enterprising, initiatives pursued by Elizabeth Carter and Elizabeth Hatchett occurred during the best part of both women's married lives. Hatchett had joined the Carters’ household in the early 1700s, not long in to her own marriage, affording up to twenty years for Carter and Hatchett to develop a working partnership. In fact, the case suggests that the relationship between these two married women was more significant than their conjugal ties for both women's material well-being and possibly also for their emotional well-being. The only occasion when either woman suffered the legal constraints of coverture was when their partnership broke down, a little over a year before Elizabeth Carter's death. It was acrimony between the two women (rather than any direct patriarchal intervention) that put an end to their successful negotiation of the opportunities offered by the fluidity between household and market; cash and goods; and the so-called ‘formal’ and ‘informal’ economy.

## Women's lending networks

The incidental details of Carter and Hatchett's lending activities confirm the impressions pieced together by Beverly Lemire of women's responsibility for managing small-scale quotidian credit transactions on behalf of their households, as well as their wider role in facilitating the credit relations of others. Women were not only active borrowers in their own right, they also commonly acted as guarantors for others, overseeing the process of converting assets into credit and cash to facilitate a growing density of exchange.^20^ Seven out of the eleven witnesses who detailed their own borrowing from Carter or Hatchett were women, all of whom, barring one widow, were married. The sums involved were relatively small, ranging from 20s (£1) to £30, and the majority of the loans actually specified by witnesses amounted to £10 or less. Some such instances were apparently one-off transactions. Carter's sister in law (married to a labourer) recounted borrowing 20s from Hatchett ‘towards fitting out her son to be an Apprentice’, offering a silver spoon and a silver salt as a pledge.^21^ However, other witnesses detailed a longer history both of their own and of others’ borrowing, so that although the individual sums involved were small, they multiplied through regular repetition. A butcher's wife, for example, who told the court that she got her living by taking in children to nurse and by selling fruit, recalled borrowing money frequently from Carter over a period of eighteen years, ‘shee being recommended…as a person who lett out money either in greater or lesse sumes at usury’.^22^

There are various ways of contextualising the amounts of money that were reportedly lent by Carter and Hatchett. The most common sum cited by witnesses in the case was £5. While small compared with the larger and more formalised loans underwritten by well-to-do single women, or by London goldsmiths during the same period, £5 nonetheless amounted to between one and two years' wages for a domestic servant in London.^23^ It was also relatively large compared with the sums detailed in cases of property crime reported in the *Proceedings of the Old Bailey* involving stolen goods that had been pawned. The twenty-three specific sums secured as loans by pawns that were detailed in the *Proceedings* in the five-year period between 1718 and 1722 ranged from 6 Guineas for a watch to 3s 6d for a drugget coat (a coat made of heavy wool).^24^ The size of the loans extended by Carter and Hatchett routinely exceeded the sums recounted in Old Bailey trials, suggesting that they did not solely cater to the lower end of the pawnbroking market. The women who sought Carter and Hatchett's services testified to making their livings in a variety of ways, from washing clothes, cutting wool and taking children in to nurse, to retailing and keeping a public house. Their husbands’ stated occupations included a labourer, butcher, porter, cloth-workers and a waterman, while the men who borrowed directly from Carter or Hatchett included shoemakers, a sexton, a gardener and a victualler.

The witnesses who explicitly identified themselves as borrowers were drawn from across and beyond the City of London, which also meant that Carter and Hatchett were not merely serving parochial or neighbourly needs. This was as true for their female as for their male debtors. None of their female clients lived in the same parish as Carter and Hatchett (St Stephen, Coleman Street). Three women debtors were from the neighbouring parish of St Giles Cripplegate (which was also the parish where Carter's husband, Humphrey, had been born).^25^ The others were drawn from further afield, from both within and beyond the City, including one from St Sepulchre without Newgate and two from Christ Church, Southwark.^26^ The male borrowers similarly hailed from parishes within and without the City, ranging from St Stephen Coleman Street to Battersea. The witnesses called to testify in the case also had widespread origins, including neighbours from Bell Alley (where Carter and Hatchett had lived), others drawn from a variety of parishes within the City of London and extending outwards to Shoreditch and St Anne Soho as well as to locations south of the River Thames including Stockwell and Newington. A further two witnesses were from Hayes, Middlesex, and one – the sister of a former lodger of Carter's, now married to a gentleman – had journeyed all the way from Bristol to provide evidence. Witnesses were connected to Carter and Hatchett through ties of kinship, friendship and neighbourhood, through Carter's midwifery practice (Carter had delivered the children of several deponents in the case), and through business links of various kinds, of which Carter and Hatchett's lending activities constituted a significant part.

While female-centred, the networks of credit that flowed from Carter and Hatchett were not female-specific. Two shoemakers, both of St James, Duke Place, admitted borrowing money from both women, along with a waterman from St Martin Orgar.^27^ Richard Harris, a gardener from Battersea, first encountered Carter when standing surety for a loan of £5 which she extended to a carpenter who worked for Harris, after which point Harris also borrowed money from Carter ‘as hee sometimes wanted it himselfe in his way of trade’.^28^ Some of the men who borrowed from Carter and Hatchett were nonetheless linked to women borrowers who had secured the necessary introduction. The sexton of St Stephen Coleman Street, who was Carter and Hatchett's neighbour, recounted his mother borrowing £10 on several occasions from Hatchett, who a few years later was followed by his brother who regularly borrowed the smaller sum of 40s.^29^

The men as well as the women who had acquired cash from Carter and Hatchett spoke of their ‘intimacy’ with the two women, suggesting that their credit relations were woven into and generated broader networks of mixed sociability. Verifying his knowledge of Carter over a period of fifteen or sixteen years, a shoemaker claimed that through his frequent borrowing from her he contracted ‘an intimate friendshipp & acquaintance’ on account of which he had visited her once or twice a week.^30^ Carter and Hatchett's lending activities *might* therefore be treated as an extension of the ties of reciprocity that secured the informal credit relations that were routinely brokered by women, and that in some circumstances represented mutual aid rather than enterprise. One witness even spoke of Carter herself seeking a loan when in straightened circumstances not long before her death. At this point, Carter reportedly approached Christiana James (the wife of one of Hatchett's former debtors), claiming that she had ‘not a farthing to help herself’, borrowing 5s from Christiana and ‘begging’ Christiana's husband to put his hand to a note for 40s, which loan Carter promised to pay off in weekly instalments.^31^ However, extensions of ‘friendship’ also carried connotations of patronage and established webs of dependency.^32^ When Carter and Hatchett ‘let’ their money out to use, they provided their ‘friends’ with a service which generated ties of obligation, and for which they also expected a good return.

While often flowing through channels of so-called ‘friendship’ and ‘intimacy’, Carter and Hatchett's business activities were clearly commercially driven. Their dealings do not fit the ‘pattern of unassuming enterprise’ that, according to Lemire, characterised women's lending, but were larger scale and more profit oriented.^33^ According to one witness, Hatchett had borrowed £300 to set herself up in ‘Employment’ as a moneylender, and she was reputed to have grown rich from extending loans in return for pawns. Carter was described by several witnesses as directing a large-scale operation, with another of Carter's sisters claiming to have seen ‘money lye by her in heaps in order to bee lent out’.^34^ More often, and more importantly, witnesses described the many material markers of wealth and success on display in Carter's household and on her person in the form of fashionable clothes and jewellery. Carter's clothes were described in great detail by some witnesses, such as Elizabeth Perry, the wife of a clothworker, who remarked that she had gone about ‘fine & toppingly drest in good & fashionable silkes & sattins & with very good lace in her head clothes’. A widow from Christchurch, Southwark, claimed that Carter ‘went as fine & as richly drest as the best merchants wife in the Citty might doe’. Sara Carter, married to Elizabeth Carter's nephew Richard (a weaver), described her aunt as having been shortly before her death decked in ‘a flower'd silke damask gowne & pettycoat, the ground whereof was yellow & the flower thereof white a very good laced suit of head clothes & ruffles & a scarlet Cloth Cloake trimmed with gold’. She also estimated that a ‘crotchet’ (or brooch) of diamonds, also worn by Carter, had been worth £50.^35^ Others attempted to value a gold chain and a gold striking watch that had reportedly been in Carter's possession. These assessments attest to the importance of clothes not just as signifiers of status (in this case deemed ‘as fine as any merchant's wife’), but also as repositories of wealth, and as a kind of ‘alternative currency’.^36^

Witnesses also described with considerable precision the goods and furniture in Carter's possession, although those called on behalf of Eleanor Jennings stressed that it had not been Carter's own, but belonged to the lodgers to which Carter had rented rooms. Their appraisals attest to the ways in which domestic items other than clothes also functioned as repositories of wealth and a medium of exchange. Elizabeth Perry, married to a clothworker of the neighbouring parish, St Giles Cripplegate, recalled ‘good & fashionable goods & furniture’ including ‘high back armed Chaires, red Curtaines[,] two Clockes, large looking glasses in the panel & in the Chimney & other such furniture as were fit & convenient for any Gentleman to lodge in’.^37^ A former servant of Carter's itemised her household goods with remarkable detail and skill. She listed ‘a great deale of plate’ including ‘two silver tankards, a silver pinte cup[,] a silver pinte mugg[,] two silver candlesticks[,] two silver coffee potts one for an ounce of Coffee & another for an ounce and a half[,] one silver porringer[,] salts, two silver salvers[,] a sett of Castors & several dozens of silver spoons’. She also estimated that, besides the furnishings in the rooms she let to tenants, Carter had possessed eight or nine beds made of cherry wood, ‘laid one upon another’ in one of the back garrets in her house. Among other items she also singled out ‘foure handsome lookeing glasses, glasses, sconces three standing Clocks, a Scrutoire [escritoire] with a great deale of China, [and] Caine Chaires’, all of which signified surroundings ‘rather fitt for a Gentleman then a Tradesman to live in’.^38^ Such knowledge was not just born of the intimacy afforded by domestic service. Several of Carter's friends, neighbours and acquaintances conjured similarly detailed descriptions of her material surroundings, with many (including her servant) assigning a cash value to particular items or to bundles of goods, using the same appraisal skills with which we are familiar from the processes undertaken to compile probate inventories.^39^

The female deponents in this case described Carter's possessions in much greater detail than their male counterparts, in ways which attested to the importance of moveable property in general as a form of investment (i.e. including, but not restricted to, clothes). This corresponds with women's use of a greater level of detail in wills to describe bequests of moveable property compared with men's, which might be attributed as much to their facility for evaluating investments as to the relative importance of women's affective ties.^40^ Household goods (like clothes) not only signified status but functioned as a repository of wealth that could also serve as a cash equivalent. The skills involved in such assessments informed a wider culture of appraisal, in which judgments about the cash value of the goods in people's possession were central to assessments and assertions of credit. In a cash-scarce economy, the vast majority of transactions were conducted on trust. Craig Muldrew has argued that, as a consequence, credit relations were brokered in a moral economy and depended heavily on assessments of reputation in ethical terms. This process, according to Muldrew, was constituted by the social circulation of ‘the self in terms of virtuous attributes’.^41^ The ways in which witnesses enumerated the ‘worth’ of the goods in their own and others’ possession are suggestive, however, of the material basis to assessments of credit and social standing. People monitored possessions as much as virtuous behaviour as the basis for decisions about the conditions of exchange. The ‘selves’ that consequently circulated were assigned a cash value. The negotiation of trust in the early modern economy was therefore built on solid material foundations.^42^

Considerable skills of appraisal informed expectations that people in early modern England could routinely place a monetary value on their ‘worth’ (which was estimated with reference to their moveable property and the extent to which they were indebted). The routine assignment of a cash value to goods and to persons suffused daily life in the early modern period. Elizabeth Carter, for example, was reported by various witnesses as having attached values to her ‘worth’ ranging from £1,500 to £18,000 at various points during her life, which self-assessments were in turn judged with varying degrees of scepticism.^43^ Especially in urban centres, where the bulk of people's worth was stored in clothes and household goods rather than in crops and livestock, intimate *knowledge* of each other's assets as well as one's own was crucial for establishing the relations of trust which underpinned the majority of exchange. This knowledge was gathered through the networks of sociability to which women were central, and it is clear that women developed considerable skills of appraisal that informed this process. The testimony of the married women in this case is further evidence of the considerable sense of entitlement they felt towards the moveable property that constituted their households (much of which they were charged with managing and protecting), as well as the skills they commanded in judging the value of the assets of others.^44^ Women's consumption therefore constituted a form of investment, rather than simply a means of display and, when approached cannily, consumption formed part of the ‘prudent economy’ expected of wives rather than its antithesis.^45^ Indeed, married women's responsibilities for saving and accounting for household resources comprised one of the few forms of women's work explicitly acknowledged by even the most conservative conduct writers concerned with delineating domestic duties.^46^ Household management was a form of asset management.

Pawnbroking, of course, depended on – and honed – precisely these skills. The author of the *London Tradesman* opined that a seven-year apprenticeship to learn the business of a pawnbroker was ‘rather too little’ to ‘become Judge of the almost infinite Number of Goods he is obliged to receive as Pledges’. This was because the trade required ‘a great deal of Judgment and Acuteness to become thoroughly Master of it’ (including being a ‘Master of Figures’).^47^ However, the skills he identified were just those that informed women's quotidian social accounting whereby they assessed each other's credit and asserted their own. Judging from their ability to assign market value to goods (expressed in terms of a cash sum), the wives charged with their households’ asset management, as well as single and widowed women, exercised ‘mastery of figures’ as well as expertise in the conditions of exchange. (This was evidently the case for the higher-ranking women who produced their own account books, but it was by no means dependent on literacy skills.)^48^ It is therefore perhaps unsurprising that women were relatively well-represented among pawnbrokers as well as among those who facilitated the extension of their services by presenting goods on others’ behalf.^49^ A search of London and Middlesex Sessions papers, as well as the *Proceedings of the Old Bailey*, for the five-year period from 1718 to 1722, turned up ninety-seven references to pawnbrokers.^50^ Of those whose gender was identified, 27 per cent were women. Women were also named as facilitators of exchange, acting as trusted intermediaries for others who wanted to realise the cash value of items in their possession.

Pawnbroking therefore represented the extension of skills which had long informed women's brokerage of credit and relations of trust, but pawnbroking also contributed to the redefinition (and narrowing) of trust, with its demand for goods as securities. *Knowledge* of goods (which might be distrained in cases of default) in such circumstances did not suffice as the basis for a loan; credit was only forthcoming through transactions that appeared to have benefited the lender more than the debtor. When details of the value of stolen items can be compared with the amount they raised as pledges (derived from the *Proceedings of the Old Bailey*), it was unusual for the lender to lend as much as half the cash value of the goods concerned, and in the majority of cases it was a good deal less than 40 per cent (see Table 1).^51^

**Table 1:** Value of loans raised on items pawned, detailed in the *Proceedings of the Old Bailey*, 1719–22

Date	Item(s)	Value	Value of loan	Loan as % of value
1720	Pistols	£5	15s	15
1720	Coat and waistcoat	£1	3s 6d	17.5
1722	Watch	£5 5s	£1 1s	21
1720	Drugget coat	10s	2s 6d	25
1722	Coat and breeches	43s	13s	30
1722	Pair of bodices; petticoat	7s	2s 6d	36
1721	Silver watch	£4	30s	38
1720	Pair of linen sheets; silver spoon	19s	7s *6*d	39
1719	Drugget coat	10s	4s	40
1721	2 silver spoons	14s	6s	43
1720	Silver cup	£9	£4 10s	50
1719	Pair of silk stockings	12s	6s	50
1722	Watch	£3	£1 15s	58

While pawnbroking may have overlapped with informal charity (when favourable terms were arranged, for example, or debts forgiven), it also represented a break from it by requiring securities from which brokers could protect themselves from loss and from which enterprising women such as Carter and Hatchett were well-placed to profit. Carter, for example, was quick to take advantage of a defaulting debtor (a porter) who had mortgaged his wife's house to Carter as security for a loan of £30. Carter swiftly took possession of the property when the porter was unable to keep up his weekly payments. His wife, who subsequently was reduced to occupying her former house as a tenant (paying Carter between £4 and £5 per annum in rent), earning her living by washing clothes and running a chandler's shop, recounted how Carter had acquired this and other houses at a price well below their market value.^52^

Both Carter and Hatchett were represented as having a sharp eye for opportunities to turn a profit and to diversify their interests. Besides lending money and storing the associated returns in clothing and household goods, witnesses described a wider range of investment strategies pursued by Carter and to a lesser extent by Hatchett. Hatchett reportedly converted some of her profits into leases on tenements by ‘purchasing estates’.^53^ Both women appear to have invested in lottery orders, with one witness describing Carter as ‘an adventurer in the state Lottery’, and another recalling Hatchett collecting the interest due on lottery orders issued in her name.^54^ Several witnesses also recounted Carter's attempts to diversify her interests (although they disagreed whether this was a sign of her wealth and enterprise or of her search for expedients in the face of poverty). In 1721, Carter had invested in a tobacco cutting engine and a hogshead of tobacco, which she intended to sell in customised papers that had been printed to bear the brand ‘Carter's Best Virginia’. One of her nephews also testified that she had enquired about the costs required to set herself up in the business of ‘Engine weaving’.^55^

Despite the fact that she was married for the bulk of the period which was being described, witnesses clearly assigned ownership of the goods they detailed to Carter, and credited her with considerable ability to generate income. Although part of Mary Lucas's strategy in attempting to secure any goods left by her deceased sister was to emphasise that Carter had been left a wealthy widow by Humphrey Carter (who reportedly had left off his trade as a baker having somewhat implausibly made a fortune sufficient to live like a gentleman), this was a relatively minor part of the stories told by Lucas's witnesses.^56^ They instead predominantly ascribed Carter's wealth to her *own* enterprise and initiative. In part, they linked her riches to her proficiency as an ‘eminent & experienced’ midwife, enjoying a long career ministering to ‘persons of good fashion & credit’.^57^ Mostly, however, the descriptions of Carter's clothes, jewels and furniture were designed to signal her resourcefulness as a businesswoman and her consequent capacity for capital accumulation. Although sharing very different motivations, Jennings's witnesses also positioned Carter as the dominant partner in her marriage to Humphrey. Claiming that he had been forced to leave off his trade because he was unable to repay his accumulated debts, they implied that he had been dependent upon his wife for his maintenance. Carter had reportedly complained that her husband had ‘run out many scores of pounds’ and that had he continued to trade she would have had no bread to eat, not least since her gains would be entirely sunk in supporting his losses.^58^ The retrospective accounting of Elizabeth Carter's goods, as well as Elizabeth Hatchett's right to them, represented both women as enterprising and active agents, exercising direct claims to the fruits of their own and each other's labour.

## Married women, coverture and the early modern economy

Both women's husbands were represented as peripheral to their dealings. Hatchett lived apart from her husband, Alexander, for the best part of two decades. He constituted a shadowy figure in the case, described as a journeyman shoemaker in poor and miserable circumstances (albeit in response to a question that sought to establish that Hatchett had no means other than the wages paid to her by the Carters on which to live). One witness claimed that Alexander Hatchett had been reduced to poverty on account of his ‘vicious life’, which had rendered his one-time position as a master shoemaker unsustainable.^59^ Several others confirmed that he had been confined in Wood Street Counter (a debtor's prison) for at least two years before Carter's death.

However, it appears that Alexander Hatchett's *name* was used in Hatchett's and possibly Carter's dealings in notes provided to secure their loans. One witness remarked that Carter and Hatchett ‘let out money on Pawns in partnership in the Names of other People’.^60^ In addition, when a shoemaker borrowed money from Elizabeth Carter he secured his loan by notes made payable to Alexander Hatchett (which notes were subsequently in the possession of Eleanor Jennings, which she then destroyed in order to issue Hope a fresh note whereby she became his creditor).^61^ Alexander Hatchett was also named in a trial heard at the Old Bailey as one of the victims of a theft involving the goods of Elizabeth Carter.^62^ Whether Alexander Hatchett was aware of the use of his name to secure his wife's lending activities is unclear. Caesar Shuttleworth, a victualler in St Giles Cripplegate, and one of the witnesses who had been close to both Carter and Hatchett (named as a beneficiary in the latter's will), testified that he thought Alexander Hatchett had been imprisoned by a ‘sham Action’ at Elizabeth Hatchett's connivance.^63^ Several witnesses deposed that Alexander Hatchett had been released from Wood Street Counter shortly after Carter's death, sporting a new suit of clothes. Shuttleworth, who went with Hatchett to release her husband, revealed that his freedom was granted by Hatchett on condition that Alexander ‘executed a Deed whereby he barred himself from meddling with any of his Wifes effects upon Condition that she should give him a new Suit of Cloaths’, which Shuttleworth valued at 23s.^64^

Alexander Hatchett's was not the only name used in the promissory notes in Carter's and Hatchett's possession. The leases to several tenements were purchased by Carter using her widowed mother's name, which she also used to secure loans in return for pawns. Carter's mother had also drawn up a letter of attorney to empower Hatchett to receive rents due to her from various tenants.^65^ Elizabeth Carter and Elizabeth Hatchett's partnership broke down in 1719, when Hatchett faced two prosecutions in the Old Bailey for stealing Carter's goods – which charges the court deemed to have been maliciously instigated by Carter, without justification.^66^ At this point, both women placed newspaper advertisements attempting to recover their debts and to ensure that funds out on loan did not end up in each other's hands. These advertisements also named a ‘Mr Joseph Batt’ as a party in Carter and Hatchett's dealings.^67^

Besides calling on the services of many proxies, it may well be that (when functional) Carter and Hatchett's partnership had also protected them from some of the constraints of coverture they faced as married women. By remaining flexible about to whom debtors were legally obliged, they sidestepped the claims of their husbands to their earnings, but also (and perhaps more importantly) any claims of their husbands' *creditors.* Their negotiation of their legal position was not necessarily, therefore, craftily connived resistance to the patriarchal proscription of married women's property rights, but may have been a means of protecting their earnings from their husbands’ liabilities. Both men were described as being heavily in debt. While it is clear that some married women used proxies and other devices to sidestep the constraints of marital property law to their sole advantage (without the knowledge of their husbands), such negotiations could also serve the mutual interest of couples. These kinds of devices were undoubtedly part of the culture of popular legalism that could be exploited by spouses working together as well as manipulated by women alone in attempts to reduce their legal disadvantage.

Both dynamics appear to have been at work in this case. Whether or not their business dealings were fully endorsed by their husbands, Carter and Hatchett clearly enjoyed considerable latitude in relation to their spouses. According to witnesses in the testamentary dispute, Carter and Hatchett's working partnership had – at its height – apparently taken precedence over their husbands’ claims, in terms of the intimacy associated with it as well as any material obligations to their spouses. Supporting Eleanor Jennings's claims that Hatchett had rightfully inherited Carter's goods, a former lodger in Carter's house deposed that Hatchett and Carter had ‘had a greater Love & kindness to & for each other than one Sister could have for another. And that they had solemnly protested before God that the longer Liver of them should have all that they were worth or to that effect.’ Another witness (married to a gentleman), declared that she ‘never saw more sincere Friendship & Affection between any two Persons then there appeared to be' between Carter and Hatchett, and also spoke of their agreement that ‘the longer Liver of them should enjoy all that they had’. This agreement had been made while Humphrey Carter was still alive, with Elizabeth Carter promising her husband that Hatchett would ‘take care to maintain him’ should Elizabeth Carter die first.^68^ The witnesses concerned appeared in no doubt about which was the more important bond.

The professed intimacy between these two women, and the mutual interests it served, did not last, however. Paradoxically, it was only when their partnership broke down that coverture explicitly came into play. The newspaper advertisements placed by Carter and Hatchett around the time of Hatchett's trial for theft at the Old Bailey suggest that Humphrey Carter was quick to protect his own interests by claiming his right to his wife's property and by denying Elizabeth Hatchett's rights to it. In March 1719, Carter placed a notice claiming that she had been robbed by Hatchett and requesting repayment of all the loans on notes she had in her possession.^69^ Two weeks later, a correction was printed, declaring the contents of Carter's notice to be false, the prosecution malicious, and Hatchett was redescribed as ‘no Servant but a Partner in Trade’.^70^ (The record of the Old Bailey trial similarly reports that several witnesses ‘made it appear very plain’ that Carter and Hatchett were partners, sending out money ‘in small Parcels’ in return for pawns, adding that ‘Mrs. Hatchet was the chief Manager’.^71^) This newspaper notice ended with the assertion that Elizabeth Carter was a *feme couvert*, adding that Humphrey Carter had issued a general release to Hatchett, and had since absconded. A further advertisement advised debtors not to pay any money to Elizabeth Carter but to apply themselves to Hatchett at the Oxford Bank in Forestreet, and Hatchett also offered a reward of 2 Guineas to anyone who could give notice of the whereabouts of Humphrey Carter so that a process at law could be served on him.^72^ The balance between solidarity and strategy had clearly been tipped at this point. Any slipperiness about what belonged to whom no longer served any party's interests, in ways which invoked the blurred boundary between exchange and theft as well as the fuzzy distinction between possession and ownership that plagued marital property law.

There was a good deal more at work here, then, than coverture, which itself appears to have had a very selective, and not entirely debilitating, impact on Carter and Hatchett's enterprise. Concepts of coverture certainly did not prevent witnesses in this case from attributing skill, enterprise, resourcefulness and esteem to two married women working in partnership in the early eighteenth century, on the basis of which they amassed at the very least goods worth fighting an extensive legal battle over in court. The only point at which Carter and Hatchett appeared to have suffered coverture as a major hindrance was when their own relationship of trust broke down, at which point Humphrey Carter invoked his rights over his wife's property in order to protect himself, and possibly his wife, from the fall-out. It is likely that Elizabeth Carter's manipulation of the law of coverture was of benefit to her husband, whereas Elizabeth Hatchett apparently managed to distance herself successfully from any claims her husband might have had on her estate.^73^ It is interesting that she chose a young single woman (Eleanor Jennings) from among her neighbours as her executrix and the beneficiary of her will, clearly in the expectation that Jennings would continue her business after her death.^74^ While some of these actions are indeed best interpreted as acts of female resistance, they also attest to the routine *centrality* of married women to the commercial lives of their communities, in terms of the roles they played in relations of exchange.

It is perhaps unsurprising that witnesses who described the intimacy between Hatchett and Carter in positive terms emphasised its sisterly quality, given that such an analogy placed Hatchett's claims to Carter's goods on a comparable footing with those of Carter's actual sister, Mary Lucas, the plaintiff in the cause. However, the appeal to sisterly bonds in this case, as well as the echo of conjugal obligation attributed to Carter and Hatchett's relationship, serves as a reminder that legally cemented partnerships between sisters, as well as between unrelated women, could rival and did indeed sometimes compete with the conventions established by marital property law.^75^

These women were not simply serving their neighbourhood by offering small-scale loans inspired by the pragmatics of mutual reciprocity. They sought to turn a profit and amass a fortune, responding deftly and strategically to the range of opportunities available in a rapidly expanding and diversifying metropolis. This is not to argue that they operated on a level playing field, either with their husbands or with other men. But they were clearly central players nonetheless, not incapable of out-manoeuvring male as well as female competitors and not unwilling to take advantage of others’ misfortune. While this case is exceptional in its level of detail, and may have involved the activities of two exceptional women, it is also worth noting that not a single witness implied that Carter and Hatchett's enterprise was out of the ordinary. If Carter's own reported estimates of her fortune at the height of her achievements are even part-way credible, she may have enjoyed comparable success to Elizabeth Waltears, a London pawnbroker who insured her business for £1,000 with the Royal Exchange Insurance Company in 1734, and would have well-exceeded the business capacity of a Rotherhithe widow, Ann Tosler, who insured goods in pledge, her stock in trade and household wares to the value of £200 in 1740.^76^

Yet the activities detailed in this case might also speak to the quotidian experiences of women in early modern England, especially in terms of the borrowing they undertook, and the associated management of household resources. Women's responsibilities for asset management, that began with singlewomen's investment of their portions, did not end at marriage but took on a new dimension as the assets in their possession were largely converted to the stock of goods that underpinned their households’ ability to negotiate credit. Women's responsibility for their households’ ‘stuff’ did not consign them to a domestic sphere, somehow detached from a commercial economy, but actually enabled that economy to function. We might liken them to bankers, and should not be surprised to discover that they sought to do as much as possible to maximise their investments.^77^ Their consumption strategies served processes of saving and accumulation rather than simply fulfilled desires for comfort, emulation or display. The consequent asset management, which in an urban context like London some women were able to pursue on an extensive and commercially oriented scale, was, therefore, another form of married women's work which can be added to our growing appreciation of female enterprise as well as industriousness in a rapidly developing economy.

## Footnotes

1 Elizabeth C. Sanderson, Women and Work in Eighteenth-Century Edinburgh (Basingstoke, 1996); Hannah Barker, The Business of Women: Female Enterprise and Urban Development in Northern England 1760–1830 (Oxford, 2006); Nicola Phillips, Women in Business 1700–1850 (Woodbridge, 2006); Christine Wiskin, ‘Businesswomen and Financial Management: Three Eighteenth-Century Case Studies’, Accounting, Business and Financial History, 16, 2 (2006), 143–61; Amy Louise Erickson, ‘Married Women's Occupations in Eighteenth-Century London’, Continuity and Change, 23 (2008), 267–307; Amy Louise Erickson, ‘Eleanor Mosley and Other Milliners in the City of London Companies 1700–1750’, History Workshop Journal, 71 (2011), 147–72; Deborah Simonton and Anne Montenach, eds., *Female Agency in the Urban Economy: Gender in European Towns, 1640–1830* (2013); Maxine Berg, ‘What Difference Did Women's Work Make to the Industrial Revolution?’, History Workshop, 35 (1993), 22–44.

2 Jan de Vries, ‘The Industrial Revolution and the Industrious Revolution’, Journal of Economic History, 54, 2 (1994), 249–70; Jan de Vries, The Industrious Revolution: Consumer Behaviour and the Household Economy, 1650 to the Present (Cambridge, 2008).

3 The classic statement linking eighteenth-century consumption to social emulation is Neil McKendrick, ‘The Consumer Revolution of Eighteenth-Century England’, in *The Birth of a Consumer Society: The Commercialization of Eighteenth-Century England*, ed. Neil McKendrick, John Brewer and J. H. Plumb (1982). For alternative explanation of novel consumption patterns, see especially Lorna Weatherill, *Consumer Behaviour and Material Culture in Britain 1660–1760* (1988); Carole Shammas, The Pre-Industrial Consumer in England and America (Oxford, 1990); Beverley Lemire, Fashion's Favourite: The Cotton Trade and the Consumer in Britain 1660–1800 (Oxford, 1991); Maxine Berg, ‘Women's Consumption and the Industrial Classes of Eighteenth-Century England’, Journal of Social History, 30, 2 (1996), 415–34.

4 Craig Muldrew, ‘“A Mutual Assent of Her Mind”? Women, Debt Litigation and Contract in Early Modern England’, History Workshop Journal, 55 (2003), 47–71.

5 See especially B. A. Holderness, ‘Credit in a Rural Community, 1660–1800: Some Neglected Aspects of Probate Inventories’, Midland History, 3 (1975), 94–115; Amy Froide, Never Married: Singlewomen in Early Modern England (Oxford, 2005), 130–7; Judith Spicksley, ‘“Fly with a Duck in thy Mouth”: Single Women as Sources of Credit in Seventeenth-Century England’, Social History, 32 (2007), 187–207; Judith Spicksley, ‘Usury Legislation, Cash and Credit: The Development of the Female Investor in the Late Tudor and Stuart Periods’, Economic History Review, 61 (2008), 277–301.

6 Amy Louise Erickson, ‘Coverture and Capitalism’, History Workshop Journal, 59 (2005), 1–16.

7 Marjorie Keniston McIntosh, ‘Women, Credit, and Family Relationships in England, 1300–1620’, Journal of Family History, 30 (2005), 143–63. On the limited cash supply in the early modern economy, see Craig Muldrew, The Economy of Obligation: The Culture of Credit and Social Relations in Early Modern England (Basingstoke, 1998); Craig Muldrew, ‘“Hard Food for Midas”: Cash and its Social Value in Early Modern England’, Past and Present, 170 (2001), 78–120.

8 On married women's authority, see especially Laura Gowing, Common Bodies: Women, Touch and Power in Seventeeth-Century England (New Haven and London, 2003).

9 Linda Pollock, ‘Childbearing and Female Bonding in Early Modern England’, Social History, 22, 3 (1997), 286–30; Laura Gowing, ‘Secret Births and Infanticide in Seventeenth-Century England’, Past and Present, 156 (1997), 87–115; Laura Gowing, ‘Ordering the Body: Illegitimacy and Female Authority in Seventeenth-Century England’, in Negotiating Power in Early Modern Society: Order, Hierarchy and Subordination in Britain and Ireland, ed. Michael J. Braddick and John Walter (Cambridge, 2001).

10 Tim Stretton, ‘The Legal Identity of Married Women in England and Europe 1500–1700’, in Europa und seine Regionen: 2000 Jahre europäische Rechtsgeschichte, ed. Andreas Bauer and Karl H. L. Welker (Cologne, 2006). On the complex implications of coverture, see Margot Finn, ‘Women, Consumption and Coverture in England, *c*. 1760–1860’, Historical Journal, 39 (1996), 703–22; Joanne Bailey, ‘Favoured or Oppressed? Married Women, Property and “Coverture” in England, 1660–1800’, Continuity and Change, 17 (2002), 351–721738782610.1017/s0268416002004253; Cordelia Beattie and Matthew Frank Stevens, eds., Married Women and the Law in Premodern Northwest Europe (Woodbridge, 2013); Tim Stretton and Krista J. Kesselring, eds., Married Women and the Law: Coverture in England and in the Common Law World (Montreal and Kingston, 2013).

11 For the characterisation of women's agency principally in terms of resistance, see especially Bernard Capp, When Gossips Meet: Women, Family, and Neighbourhood in Early Modern England (Oxford, 2003).

12 On spousal cooperation, see the Special Issue of *Continuity and Change*, 23, 2 (2008); Ariadne Schmidt, ‘The Profits of Unpaid Work. “Assisting Labour” of Women in the Early Modern Urban Dutch Economy’, History of the Family, 19, 3 (2014), 301–22.

13 London Metropolitan Archives (LMA), St Stephen Coleman Street, Rough Register of Deaths, 1711–23, P69/STE1/A/009/MS04455.

14 LMA, P69/STE1/A/009/MS04455.

15 For Elizabeth Hatchett's will, see The National Archives (TNA), PROB 11/591/465. There is no surviving copy of Elizabeth Carter's will, but Elizabeth Hatchett was named as executrix on 10 Oct. 1722, LMA, DL/C/B/001/MS 09168/034.

16 This sample is contained within the larger dataset, ‘The “Worth” of Witnesses in the English Church Courts, 1550–1728’, available from the UK Data Archive. The compilation of this dataset was funded by a research grant from the Economic and Social Research Council (RES-000–23–1111) and completed with the assistance of Dr Judith Spicksley, for which I am extremely grateful. For the character of litigation involving women, see David Lemmings, ‘Women's Property, Popular Cultures, and the Consistory Court of London in the Eighteenth Century’, in Women, Property, and the Letters of the Law in Early Modern England, ed. Nancy E. Wright, Margaret W. Ferguson and A. R. Buck (Toronto, 2004).

17 Elizabeth Carter appears to have been childless. An ‘Allexander’, son of Alexander Hatchett, was buried on 21 May 1702 in St Giles Cripplegate and was most likely the son of Alexander and Elizabeth Hatchett, who had married in St Sepulchre on 13 Apr. 1699. LMA, P69/GIS/A/002/MS06419/012, P69/SEP/A/001/MS07219/002. One witness claimed that when Hatchett joined the Carter's household, Hatchett ‘had a Child to keep’, on account of which Carter ‘tooke her in out of Charity’. LMA, DL/C/B/045/MS 09065A/012/001, fo. 469v.

18 The judgment in the case awarded goods to the value of £30 to Mary Lucas, Carter's sister-in-law, and £30 towards her expenses. Lucas's account of her expenses amounted to £60 8s 2d, which meant that the judgment constituted a net loss for Lucas of 8s 2d: LMA, DL/C/A/002/MS 09065F/004, 4 Dec. 1725, DL/C/B/MS 09185/005, 13 Dec. 1725. For a critique of historians’ attempts to discover truth in witness depositions, see Frances E. Dolan, True Relations: Reading, Literature, and Evidence in Seventeenth-Century England (Philadelphia, 2013).

19 LMA, DL/C/B/045/MS 09065A/012/001, fos. 379–501.

20 Beverly Lemire, ‘Petty Pawns and Informal Lending: Gender and the Transformation of Small-Scale Credit in England, *circa* 1600–1800', in From Family Firms to Corporate Capitalism: Essays in Business and Industrial History in Honour of Peter Mathias, ed. Kristine Bruland and Patrick O'Brien (Oxford, 1998); Beverly Lemire, The Business of Everyday Life: Gender, Practice and Social Politics in England, c. 1600–1900 (Manchester, 2005).

21 LMA, DL/C/B/045/MS 09065A/012/001, fo. 391.

22 LMA, DL/C/B/045/MS 09065A/012/001, fo. 369.

23 It was alleged in this case that Hatchett had been paid 50s (£2 10s) annually to serve as the Carters’ servant. On domestic servants’ wages, see Peter Earle, *A City Full of People: Men and Women of London 1650–1750* (1994), 125. On goldsmith bankers, see D. M. Mitchell, ‘“Mr. Fowle Pray Pay the Washwoman”: The Trade of a London Goldsmith Banker, 1660–1692’, Business and Economic History, 23, 1 (1994), 27–38; D. M. Mitchell, ed., Goldsmiths, Silversmiths and Bankers: Innovation and the Transfer of Skill, 1550–1750 (Stroud, 1995).

24 *The Proceedings of the Old Bailey Online* (*OBO*), www.oldbaileyonline.org (last accessed July 2014).

25 Humphrey Carter, son of a wire dresser, was baptised on 6 June 1669. LMA, P69/GIS/A/002/MS06419/007. I have been unable to find a record of Elizabeth and Humphrey Carter's marriage, or of Elizabeth Carter's birth (née Everett).

26 Another female borrower was from St Martin, Canon Street. Several witnesses referred to other borrowers, whose residence was not detailed.

27 LMA, DL/C/B/045/MS 09065A/012/001, fos. 465, 473, 487.

28 LMA, DL/C/B/045/MS 09065A/012/001, fo. 418.

29 LMA, DL/C/B/045/MS 09065A/012/001, fo. 397v.

30 LMA, DL/C/B/045/MS 09065A/012/001, fo. 465. On the routine description of clients as ‘friends’ in the eighteenth century, see Barker, *Business of Women*, 82.

31 LMA, DL/C/B/045/MS 09065A/012/001, fo. 393.

32 On the multi-layered concept of friendship in early modern England, see Naomi Tadmor, Family and Friends in Eighteenth-Century England: Household, Kinship, and Patronage (Cambridge, 2001).

33 Lemire, ‘Petty Pawns and Informal Lending’, 123.

34 LMA, DL/C/B/045/MS 09065A/012/001, fo. 429v.

35 LMA, DL/C/B/045/MS 09065A/012/001, fos. 427v, 434, 446.

36 Beverly Lemire, ‘Consumerism in Preindustrial and Early Industrial England: The Trade in Secondhand Clothes’, Journal of British Studies, 27 (1988), 1–24; Beverly Lemire, ‘Shifting Currency: The Culture and Economy of the Second Hand Trade in England, *c*. 1600–1850’, in *Old Clothes, New Looks: Second Hand Fashion*, ed. Alexandra Palmer and Hazel Clark (2004). See also Sanderson, *Women and Work*, 150–7. Carter was also reported by one witness to have traded in second hand clothes, LMA, DL/C/B/045/MS 09065A/012/001, fo. 441v.

37 LMA, DL/C/B/045/MS 09065A/012/001, fo. 427v. On the material markers of gentility, see H. R. French, The Middle Sort of People in Provincial England 1600–1750 (Oxford, 2007), ch. 3.

38 LMA, DL/C/B/045/MS 09065A/012/001, fo. 446r–v.

39 On probate inventories, see especially Weatherill, *Consumer Behaviour*; Tom Arkell, Nesta Evans and Nigel Goose, eds., When Death Do Us Part: Understanding and Interpreting the Probate Records of Early Modern England (Oxford, 2000), pt I.

40 Berg, ‘Women's Consumption’.

41 Muldrew, *Economy of Obligation*, 156.

42 Alexandra Shepard and Judith Spicksley, ‘Worth, Age and Social Status in Early Modern England’, Economic History Review, 64 (2011), 493–530; Alexandra Shepard, Accounting for Oneself: Worth, Status and the Social Order in Early Modern England (Oxford, 2015).

43 See, e.g., LMA, DL/C/B/045/MS 09065A/012/001, fos. 427v, 429v, 433v.

44 Bailey, ‘Favoured or Oppressed?’; Amy Louise Erickson, ‘Possession – and the Other One-Tenth of the Law: Assessing Women's Ownership and Economic Roles in Early Modern England’, Women's History Review, 16 (2007), 369–85; Alexandra Shepard, ‘The Worth of Married Women in the English Church Courts, *c*. 1550–1730’, in *Married Women*, ed. Beattie and Stevens.

45 On women's consumption as a form of work, see Jane Whittle and Elizabeth Griffiths, Consumption and Gender in the Early Seventeenth-Century Household: The World of Alice Le Strange (Oxford, 2012). On the indistinct relationship between consumption and saving in the early modern period, see Shepard, *Accounting for Oneself.* On ‘prudent economy’, see Amanda Vickery, The Gentleman's Daughter: Women's Lives in Georgian England (New Haven, 1998), ch. 4. See also Alexandra Shepard, ‘Crediting Women in the Early Modern English Economy’, History Workshop Journal, 79 (2015), 1–24.

46 E.g., *The Gentlewoman's Companion: or, A Guide to the Female Sex* (1675), attributed to Hannah Woolley, included careful money management as one of the principal duties of wives, and cautioned against ‘being too lavish’ in expenditure while remaining vigilant against servants spoiling household goods by their negligence, 107. While Woolley dismissed the ‘employment’ required to ‘govern an House’ as ‘not difficult’, she also advised that ‘the active vigilance of a good and careful Wife is the ready way to enrich a bad Husband’, 108.

47 R. Campbell, *The London Tradesman* (1747), 296–7.

48 For examples, see Judith Spicksley, ed., The Business and Household Accounts of Joyce Jeffreys, Spinster of Hereford, Records of Social and Economic History, New Series, 41 (Oxford, 2012); Whittle and Griffiths, *Consumption and Gender.* See also Keith Thomas, ‘Numeracy in Early Modern England’, Transactions of the Royal Historical Society, 37 (1987), 103–32; Rebecca Elisabeth Connor, *Women Accounting, and Narrative: Keeping Books in Eighteenth-Century England* (2004).

49 Peter Earle, *The Making of the English Middle Class: Business, Society and Family Life in London, 1660–1730* (1989), 170; Margaret R. Hunt, The Middling Sort: Commerce, Gender and the Family in England, 1680–1780 (Berkeley, 1996), 132–4.

50 ‘London Lives 1690 to 1800: Crime, Poverty and Social Policy in the Metropolis’, www.londonlives.org (last accessed July 2014).

51 See also Peter Earle, *Making of the English Middle Class*, 50. For a contemporary assessment of the interest accrued on loans secured with pledges, as well as the relative value of loans to pledges, see *An Apology for the Business of Pawn-Broking* (1744).

52 LMA, DL/C/B/045/MS 09065A/012/001, fos. 453–4.

53 LMA, DL/C/B/045/MS 09065A/012/001, fo. 390.

54 LMA, DL/C/B/045/MS 09065A/012/001, fo. 448. On women's more formal investment practices in the eighteenth century, see Ann M. Carlos, Karen Maguire and Larry Neal, ‘Women in the City: Financial Acumen during the South Sea Bubble’; Anne Laurence, ‘Women, Banks and the Securities Market in Early Eighteenth-Century England’; Christine Wiskin, ‘Accounting for Business: Financial Management in the Eighteenth Century’, all in *Women and their Money 1700–1950*, ed. Anne Laurence, Josephine Maltby and Janette Rutterford (2009).

55 LMA, DL/C/B/045/MS 09065A/012/001, fos. 461, 407.

56 Humphrey Carter was assessed (on rent) at between £1 8s 0d and £3 4s in the Land Tax records between 1702 and 1720: LMA CLC/525/MS 11316/013–064. Carter also attended the vestry meetings of St Stephen Coleman Street between 1712 and 1718, and served as a questman in 1718, but he did not hold any other parish office. LMA P69/STE1/B/001/MS 04458/001/002.

57 LMA, DL/C/B/045/MS 09065A/012/001, fos. 421v, 416.

58 LMA, DL/C/B/045/MS 09065A/012/001, fo. 485v.

59 LMA, DL/C/B/045/MS 09065A/012/001, fo. 485v.

60 LMA, DL/C/B/045/MS 09065A/012/001, fo. 397v.

61 LMA, DL/C/B/045/MS 09065A/012/001, fo. 466v.

62 The case was brought against four men for perverting the cause of justice, *OBO*, t17221205–43.

63 LMA, DL/C/B/045/MS 09065A/012/001, fo. 496.

64 LMA, DL/C/B/045/MS 09065A/012/001, fo. 496v.

65 LMA, DL/C/B/045/MS 09065A/012/001, fo. 478r–v. This claim was denied by Mary Lucas in the second personal answer she supplied in the case, DL/C/B/047/MS 09065D, fo. 213.

66 *OBO*, t17190408–8, t17191204–43. Hatchett was acquitted on both occasions.

67 *Post Man and the Historical Account*, 7–10 and 10–12 Mar., 24–6 Mar., 14 Apr. 1719.

68 LMA, DL/C/B/045/MS 09065A/012/001, fos. 485, 491.

69 *Post Man and the Historical Account*, 10–12 Mar.

70 *Ibid*., 24–6 Mar.

71 *OBO*, t17190408–8.

72 *Post Man and the Historical Account*, 14 Apr. 1719.

73 Alexander Hatchett appointed proctors to represent his claims to his late wife's estate in June 1723, but the case was shortlived and clearly unsuccessful, since it was Eleanor Jennings who was the target of Mary Lucas's claims to Elizabeth Carter's goods. TNA, PROB 31/14/386. See also PROB 18/37/98, PROB 24/60, 4 July 1723.

74 Her motives for favouring Eleanor Jennings perhaps also related to the support Hatchett may have received from Eleanor Jennings's mother in relation to Hatchett's attempts to secure Carter's goods.

75 Phillips, *Women in Business*, 32–3, 169.

76 Beverly Lemire, Dress, Culture and Commerce: The English Clothing Trade before the Factory, 1660–1800 (Basingstoke, 1997), 108.

77 Shepard, ‘Crediting Women’.

